# In vitro and in vivo evaluation of iRoot BP Plus as a coronal sealing material for regenerative endodontic procedures

**DOI:** 10.1007/s00784-023-05468-3

**Published:** 2024-01-03

**Authors:** Ning Yang, Wenxiao Yang, Rou Shen, Shengcai Zhang, Tianchi Ma, Yao Liu

**Affiliations:** 1 Department of Pediatric Dentistry, School and Hospital of Stomatology, China Medical University, 117 Nanjing North Street, Shenyang, 110002 China; 2 Liaoning Provincial Key Laboratory of Oral Diseases, Shenyang, China; 3 Department of Orthodontics, Shenyang Stomatology Hospital, 138 Zhongshan Road, Shenyang, 110004 China

**Keywords:** Regenerative endodontic procedures, iRoot BP plus, Stem cells from apical papilla, Coronal sealing material, Pulp-dentin complex formation, Osteo/odontogenic differentiation

## Abstract

**Objectives:**

To investigate in vitro effects of a nanoparticle bioceramic material, iRoot BP Plus, on stem cells from apical papilla (SCAP) and in vivo capacity to induce pulp-dentin complex formation.

**Materials and methods:**

The sealing ability of iRoot BP Plus was measured via scanning electron microscopy (SEM). SCAP were isolated and treated in vitro by iRoot BP Plus conditioned medium, with mineral trioxide aggregate (MTA) conditioned medium and regular medium used as controls, respectively. Cell proliferation was assessed by BrdU labeling and MTT assay and cell migration was evaluated with wound healing and transwell assays. Osteo/odontogenic potential was evaluated by Alizarin red S staining and qPCR. Pulp-dentin complex formation in vivo was assessed by a tooth slice subcutaneous implantation model.

**Results:**

iRoot BP Plus was more tightly bonded with the dentin. There was no difference in SCAP proliferation between iRoot BP Plus and control groups (*P* > 0.05). iRoot BP Plus had a greater capacity to elevated cell migration (*P* < 0.05) and osteo/odontogenic marker expression and mineralization nodule formation of SCAP compared with MTA groups (*P* < 0.05). Furthermore, the new continuous dentine layer and pulp-like tissue was observed in the iRoot BP Plus group in vivo.

**Conclusions:**

iRoot BP Plus showed excellent sealing ability, promoted the migration and osteo/odontogenesis of SCAP and induced pulp-dentin complex formation without affecting the cell proliferation, which indicated iRoot BP Plus was a promising coronal sealing material in REPs.

**Clinical relevance:**

The coronal sealing materials play crucial roles for the outcomes of REPs. This study showed that iRoot BP Plus has good coronal sealing and promote pulp-dentin complex formation compared with MTA, providing experimental evidences for the clinical application of iRoot BP Plus as a promising coronal seal material in REPs.

**Supplementary Information:**

The online version contains supplementary material available at 10.1007/s00784-023-05468-3.

## Introduction

Immature permanent teeth that have erupted but have not reached the plane have short roots and an open apex, and continue to develop the roots after eruption. Pulp necrosis and apical periodontitis of immature permanent teeth are caused by caries, trauma, and tooth abnormalities [1]. Pulp necrosis leads to the interruption of root development and further reduces the physiological function and lifespan of affected teeth [1]. Regenerative endodontic procedures (REPs) refer to biologically based procedures designed to replace damaged structures, including dentine and root structures, as well as cells of pulp-dentine complex [2]. After implementing the REPs, continued root development and hard tissue deposition on the dentinal wall can occur under ideal circumstances. Therefore, REPs are the most desirable treatment for pulp necrosis of immature permanent teeth.

For a favorable prognosis, effective root canal disinfection, a good coronal seal, and appropriate intracanal scaffolds are critical factors for REPs [3]. Optimum coronal sealing materials must be noncytotoxic, easy to operate, chemically and mechanically stable, and osteo/odontoinductive [3]. Mineral trioxide aggregate (MTA) has been used extensively as pulp capping, root filling and repair materials, because of its biocompatibility [4, 5]; therefore, it is often regarded as the gold standard for evaluating congeneric new biomaterials [6]. However, MTA presents disadvantages such as its grainy consistency, discoloration potential, toxic components and complex operation procedures, which increase the risk of contamination [7]; thus, the clinical application of MTA in REPs is limited. Therefore, a novel ready-to-use biomaterial that does not lead to discoloration and possesses excellent biocompatibility must be developed for ideal coronal sealing in REPs.

iRoot BP Plus (Innovative Bioceramix, Vancouver, BC, Canada) is a laboratory-synthesized, premixed, ready-to-use, nanoparticulate bioceramic material developed for permanent root canal repair and surgical use [8]. The main components of iRoot BP Plus are calcium silicates and monobasic calcium phosphate, which facilitate cytocompatibility [8]. It has reported that cells exposed to iRoot BP Plus revealed a similar cytocompatibility as MTA [9–11]. Studies have shown that iRoot BP Plus could release more Si ions than MTA, which can promote the functions of stem cell [12]. In addition to material components, the material bioactivity can also affect the cellular response [13]. The excellent apatite-forming ability of iRoot BP Plus may play an important role in improving the bioactivity and biocompatibility of materials required for tissue regeneration [12]. Various in vitro and in vivo studies have demonstrated that iRoot BP Plus upregulates the expression of mineralization-related genes [12, 14], which suggests that iRoot BP Plus represents a pulp capping material. However, performing endodontic procedures on non-vital immature permanent tooth is a major challenge for clinicians. Moreover, whether iRoot BP Plus is appropriate as a coronal sealing material during REPs is not fully understood and requires further study.

The potential of REPs is in large part due to advancements in three key factors-based tissue engineering. Therefore, the cellular functions of dental stem cells that homing to root canal are crucial to the successful prognosis of REPs. Stem cells from apical papilla (SCAP) are a unique dental stem cell isolated from apical papilla tissue, which is the core tissue for root development of immature permanent tooth [15]. SCAP exhibit an elevated proliferative capacity and differentiate into odontoblasts, which play an important role in the formation of dental pulp and dentin [16]. In the apical periodontitis of immature permanent teeth, SCAP still survive and even maintain their stemness; therefore, they have been considered to be the most important source of stem cells for continued root development as well as apical closure [17]. In REPs, SCAP are homing to the root canal, which has a blood supply, and differentiate into odontoblasts, which participate in the process of pulp-dentin tissue regeneration [18]. Therefore, the biological effects of coronal sealing material on SCAP mainly determine the therapeutic outcomes of REPs, including cell viability, migration, and osteo/odontogenic differentiation of SCAP.

To the best of our knowledge, the cellular responses of SCAP under iRoot BP plus have not been reported. Therefore, this study evaluated the effects of iRoot BP Plus on the proliferation, migration, and differentiation of SACP in vitro and performed histological analyses of pulp-dentin complex regeneration in vivo.

## Materials and methods

### Isolation and characterization of SCAP

Caries-free, impacted third molars with immature roots were collected from the outpatient operating room at the Affiliated Stomatology Hospital of China Medical University. Informed consent was obtained from patients and parents (patients 15-18 years old). The experiment was approved by the Ethics Committee of the School of Stomatology, China Medical University (201315). The SCAP were harvested and cultured as previously described [19]. 4 mg/mL Dispase II (Boehringer Ingelheim, Mannheim, Germany) and 3 mg/mL collagenase I (Worthington Biochemical Co., Lakewood, CO, USA) were used to digest the apical papilla tissue. Single-cell suspensions were cultured in alpha minimum essential medium (α-MEM; HyClone, Logan, UT, USA) supplemented with 15% fetal bovine serum (FBS; MRC, Jiangsu, China), 100 U/mL penicillin-streptomycin (HyClone) and 0.1 mM L-ascorbic acid (Sigma-Aldrich, St. Louis, MO, USA) and incubated at 37℃ (5% CO_2_). Cells at passages 3-5 were used for subsequent experiments. SCAP at the 5th passages were used for multipotent differentiation capacity into neurogenesis and osteogenesis. For neurogenic differentiation, SCAP were inoculated at 4 × 10^4^ cells/well on a 24-well plate. After 1 day, the medium was exchanged with a neurogenic culture medium and cultured for 10 days. The expression of β-III Tubulin was detected by immunofluorescence staining. For osteogenic differentiation, SCAP at 70-80% confluence were cultured in an osteogenesis culture medium and changed every 2-3 days.

After 3 weeks, the cultured cells were stained with 2% Alizarin red S for mineralized nodule formation. For cell surface marker analysis, SCAP were prepared at a density of 1 × 10^6^ cells per 100 μL. Then 1 μL CD45, CD73, CD90, CD105 antibodies labelled with PE and CD34 labelled with FITC (1:100, BD Biosciences) were added to cell suspension and incubated for 60 min. The expression of each surface marker was detected by flow cytometry (Becton-Dickinson, Islandia, NY, USA).

### Sealing ability assay

Healthy premolars were obtained as discarded biological samples for orthodontic reasons. The endodontic access cavity was prepared in every premolar and the root canal orifices were located. The prepared roots were irrigated with 17% EDTA and dried with paper points. Then, the teeth were randomly divided into two experimental groups. In group A, iRoot BP Plus as capping material, and MTA as capping material in group B. After the materials were completely solidified, the teeth were cut in half longitudinally. The distance between the coronal sealing materials and root canal walls was detected using a scanning electron microscope (SEM).

### Material preparation

iRoot BP Plus and MTA (Dentsply Tulsa Dental, Tulas, OK, USA) were prepared according to the manufacturer’s instructions and placed into sterile cylinder plastic moulds (13 mm in diameter and 1 mm in height). The materials were stored at 37℃ and 100% humidity until completely solidification. After separation from the moulds, samples were incubated in 1 mL of α-MEM, (HyClone, UT, USA) for 72 h at 37℃ to produce the material extract, followed by filtration and preparation of several dilutions (undiluted, 1/2 and 1/4) of the conditioned medium supplemented with 15% FBS for use. The pH of the extracts was measured with a pH meter (INESA Scientific Instrument Co., Shanghai, China). The ratio of material surface area to medium volume was set at approximately 3 cm^2^ mL^-1^ in accordance with the guidelines of the International Organisation for Standardisation 10993 (12: 10.3.3).

### BrdU labeling assay

SCAP (2 × 10^4^ /well) were seeded on glass coverslips placed inside a 24-well plate. Cells were treated with BrdU solution (1:100) for 20 h, and DNA synthesis was detected by BrdU staining kit (Invitrogen, NY, USA) according to the manufacturer’s instructions. The percentages of BrdU-positive cells were calculated.

### Methylthiazolyldiphenyl-tetrazolium bromide (MTT) assay

SCAP were seeded into 96-well plates at a density of 5 × 10^3^ cells per well and incubated in conditioned medium. Cell proliferation was measured by an MTT assay. Briefly, at 1, 3, 5 and 7 days, the cells were rinsed with sterilized PBS and incubated with the MTT reagent (Life Technologies, NY, USA) at 37 ℃ for 4 h. Subsequently, DMSO (Sigma-Aldrich) was added to the cells to dissolve the crystals. Absorbance was detected at a wavelength of 540 nm via using a microplate reader (Tecan, Salzburg, Austria).

### Wound healing assay

SCAP were seeded at the density of 5 × 10^5^ cells per well on 6-well plates. After SCAP reached 90% confluence, a scratch in the cells was made with a 200μL pipette tip. Cell debris were removed and cell incubated in conditioned medium for 12 and 24 h, respectively. The samples were observed under a phase-contrast microscope, and 5 images per specimen were obtained for gap distance analysis by using Image J software.

### Transwell migration assay

SCAP were suspended in the upper chamber of a 24-well transwell system in FBS-free medium at a density of 1 × 10^5^ cells/ well, and 800 μL of material extracts were added to the lower chambers. After 24 h, the migrated cells were fixed with 4% paraformaldehyde and stained using 1% crystal violet. The number of migrated cells was counted under optical microscopy in 5 random fields.

### Osteo/odontogenic differentiation assay

SCAP were seeded at the density of 1 × 10^5^ cells per well into 6-well plates. After 3 days, the medium was replaced by an oosteo-/odontogenic medium and changed every 2-3 days.

### Quantitative real-time reverse-transcriptase polymerase chain reaction (qRT-PCR)

At days 7, the expression of Alkaline PhosPhatase (*ALP*) and Dentin sialophosphoprotein (*DSPP)*, was evaluated by using GoTaq® qPCR Master Mix (Promega Corporation, Madison, WI, USA) with the gene-specific primers. The gene-specific primers were as follows:

- *ALP* forward primer, 5’-CATCGCCTATCAGCTAATGCACA-3’;
- *ALP* reverse primer, 5’-ATGAGGTCCAGGCCATCCAG-3’;
- *DSPP* forward primer, 5’-CAGCCAGGCAGAAGCATGTC-3’;
- *DSPP* reverse primer, 5’-GGGCGAAGGCTCCAGAGGA-3’;
- *GAPDH* forward primer, 5’-GGCACAGTCAAGGCTGAGAATG-3’;
- *GAPDH* reverse primer, 5’-ATGGTCATGCAAGACGCCAGTA-3’.

#### Alizarin red S staining

After 28 days in osteogenic medium, the cells were fixed in 10% formalin for 15 min and stained with 2% Alizarin red S for 5 min. After three times washing with distilled water, calcium nodules were observed via using an optical microscopy and measured with ImageJ software.

### Tooth slice subcutaneous implantation model

Healthy premolars were obtained as discarded biological samples for orthodontic reasons. The roots of premolars were sectioned horizontally into 5 mm slices. In order to completely remove the pre-dentin and odontoblast layer, more than 1 mm of dentin (the whole pre-dentine and partial dentine) was carefully removed from the inner wall of root canal with a fissure bur. 17% EDTA was used to treat tooth fragments for 15 min, and then the tooth fragments were washed in deionized water for 10 min by an ultrasonic cleaner. The EDTA chelation and rinsing process was repeated for twice [19]. The tooth fragments were maintained in sterile phosphate buffered saline (PBS) with penicillin (100 U/ml) and streptomycin (100 mg/ml) at 4℃. One of the tooth slice orifices was sealed with MTA or iRoot BP Plus and then were sterilized at 120℃ for 20 min. Tooth slices were divided into 2 groups: (1) iRoot BP Plus (2) MTA. The root canal spaces of the root slices were filled with the gelatine sponge containing 5 × 10^5^ SCAP, and implanted subcutaneously on to the backs of immunodeficient mice. Each group contained five replicates. After 2 months, the samples were harvested, fixed in 4% paraformaldehyde, decalcified with ethylenediaminetetraacetic acid. 5 µm thick sections were produced to histology analysis.

### Statistical analysis

All data were expressed as mean ± standard deviation (SD) and analyzed using one-way analysis of variance (ANOVA) with SPSS 22.0. *P* < 0.05 was considered significant.

## Results

### Characterization of SCAP

SCAP are spindle-shaped cells in primary culture (Fig. S1a). In terms of the multipotent differentiation potential, SCAP expressed β-III tubulin, a neurogenic marker (Fig. S1b), and exhibited Alizarin red-positive mineralized nodules (Fig. S1c) after induction. In addition, flow cytometry analysis demonstrated that SCAP were positive for mesenchymal stem cell markers (CD73, CD90, and CD105) but negative for hematopoietic stem cell markers (CD34, and CD45) (Fig. S1d and e).

### Coronal sealing of iRoot BP Plus

**Fig. 1:**
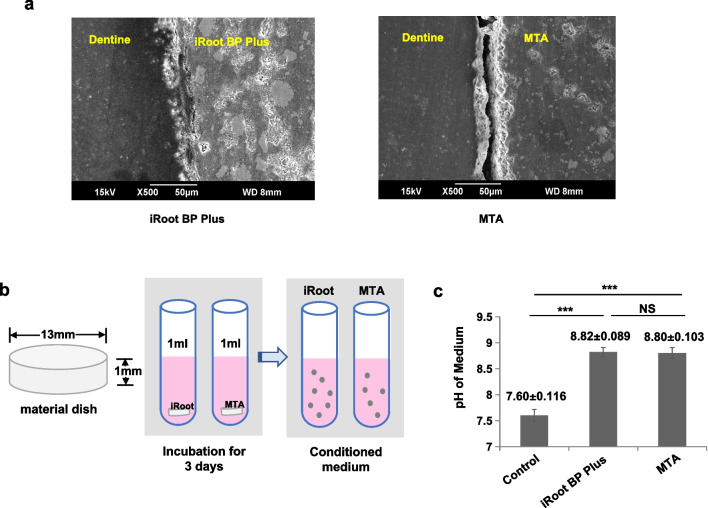
The coronal sealing and pH of iRoot BP Plus. **(a)** The SEM showed that there are only tiny cracks between iRoot BP Plus and dentin, whereas the obvious marginal gaps in the MTA group are observed. (**b**) Schema indicate the protocol for the preparation of liquid extracts of iRoot BP Plus and MTA. (**c**) The pH value of iRoot BP Plus liquid extracts was higher than that of the control group (*P* < 0.001), however, the pH values of the iRoot BP Plus and MTA groups did not significantly differ (*P* > 0.05). NS = no significant difference, Error bars: means ± SD

One of the most imperative properties that coronal filling material should have in addition to biocompatibility is sealing ability, which prevents the ingress of microorganisms and determines the success of the REPs [3]. To simulate clinical procedure of coronal filling material in REPs, 3 mm-deep iRoot BP Plus and MTA were set near the root canal orifice in our study to investigate the coronal sealing ability. A SEM examination of the coronal sealing material showed that iRoot BP Plus was tightly connected with the dentin, whereas the obvious marginal gap in the MTA group is observed (Fig. 1a). These data demonstrated that iRoot BP Plus showed excellent sealing as a coronal filling material.

### iRoot BP Plus had no effects on SCAP proliferation

In order to clarify the effects of iRoot BP Plus on the cell properties of SCAP, extraction media of iRoot BP Plus and MTA was prepared (Fig. 1b). We firstly evaluated the pH values of the three media. The pH of the iRoot BP Plus extracts was 8.82 ± 0.089, which was significantly higher than that of the control medium (7.60 ± 0.116) (*P* < 0.001); However, the pH values of the iRoot BP Plus and MTA groups did not significantly differ (*P* > 0.05) (Fig. 1c).

**Fig. 2:**
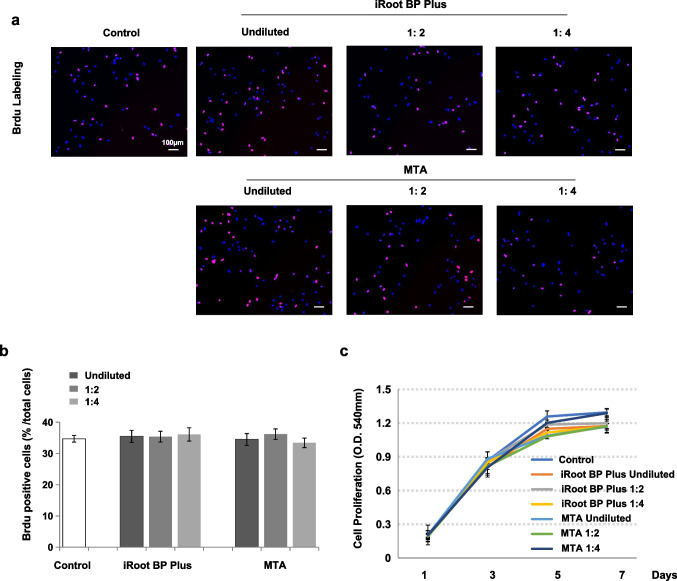
iRoot BP Plus had no effects on SCAP proliferation. **(a and b)** Brdu labeling assay showed that the cell proliferation of SCAP cultured in iRoot BP Plus conditioned medium had no significant differences when compared with the MTA and control groups (*P* > 0.05). And there were no significant differences in Brdu-positive cell ratio of SCAP among the different concentration extracts of iRoot BP Plus groups, which also seen in MTA groups (*P* > 0.05). **(c)** MTT assay showed that iRoot BP Plus conditioned medium had no significant effects on SCAP activities when compared with the MTA and control group at day 1, 3, 5 and 7 (*P* > 0.05). Error bars: means ± SD

We next prepared different dilutions (undiluted and 1/2 and 1/4 dilutions) of the conditioned medium and investigated the possible effects of the material conditioned medium on SCAP proliferation through Brdu labeling assay. The Brdu-positive cell ratio of SCAP in iRoot BP Plus group was not significantly different relative to that of the MTA group or control group (Fig. 2a and b). Moreover, significant differences were not observed in the Brdu-positive cell ratio of SCAP for the different concentration extracts of the iRoot BP Plus and MTA groups (*P* > 0.05) (Fig. 2a and b). And there were no statistically significant differences in the proliferation rates of SCAP at day 1, 3, 5, 7 among the iRoot BP Plus, MTA and control groups (*P* > 0.05) (Fig. 2c) as assessed by MTT assay. Analysis of these results suggested that iRoot BP Plus had no effects on SCAP proliferation.

### iRoot BP Plus promoted SCAP migration

**Fig. 3:**
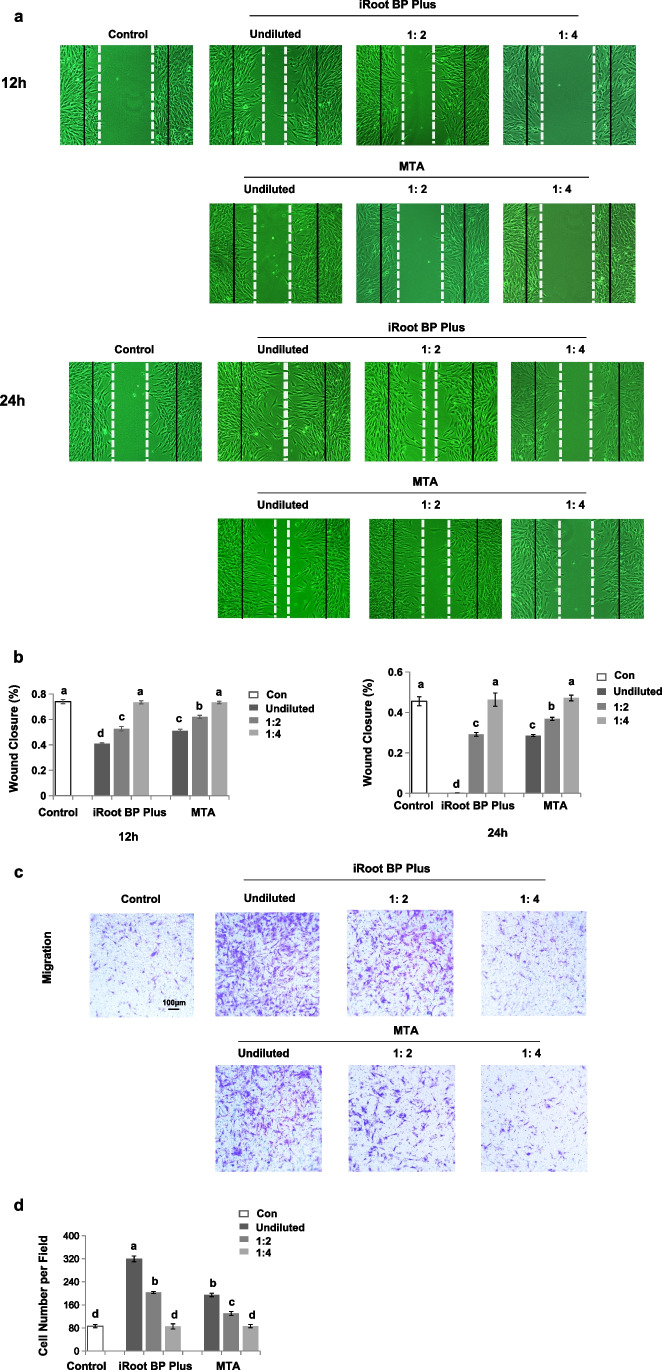
iRoot BP Plus promoted the cell migration of SCAP. (a and b) The wound healing assay showed that iRoot BP Plus had a greater capacity to elevate SCAP migration than MTA and control groups (*P* < 0.05). iRoot BP Plus significantly enhanced the wound closure percentage of SCAP in a concentration dependent manner at 12 and 24 h when compared to controls (*P* < 0.05). (c and d) Transwell cell migration assay showed that more migrated SCAP among the iRoot BP Plus treated SCAP than among the MTA treated SCAP at 36 h in a concentration dependent manner (*P* < 0.05). Error bars: means ± SD

We further explore whether iRoot BP Plus affected SCAP migration. We found that iRoot BP Plus had a greater capacity to elevate SCAP migration than the MTA and control groups (*P* < 0.05) (Fig. 3a and b). In addition, the wound closure percentage of SCAP was significantly enhanced in the iRoot BP Plus group in a concentration-dependent manner at 12 and 24 h relative to that of the controls, although a similar elevation was observed in the MTA group (*P* < 0.05) (Fig. 3a and b). The wound healing assay also showed that high doses of iRoot BP Plus (undiluted and 1/2 diluted) significantly elevated the wound closure of SCAP, especially with the undiluted solution (*P* < 0.05). Furthermore, the low dose of iRoot BP Plus (1/4 diluted) did not have a significant effect on the migration of SCAP (*P* > 0.05) (Fig. 3a and b). High doses of MTA (undiluted and 1/2 diluted) also improved the wound closure of SCAP (*P* < 0.05), while the low dose (1/4 diluted) showed a similar migration ability as the control (*P* > 0.05) (Fig. 3a and b). The result was also confirmed by subsequent transwell assays. At 24 and 36 h, there were more migrated SCAP among the iRoot BP Plus treated SCAP than among the MTA treated SCAP in a concentration dependent manner (*P* < 0.05) (Fig. 3c and d; Fig. S2a and b). Therefore, these data indicated that iRoot BP Plus significantly elevated SCAP migration in vitro.

### iRoot BP Plus improved the osteo-/odontogenic differentiation of SCAP

**Fig. 4:**
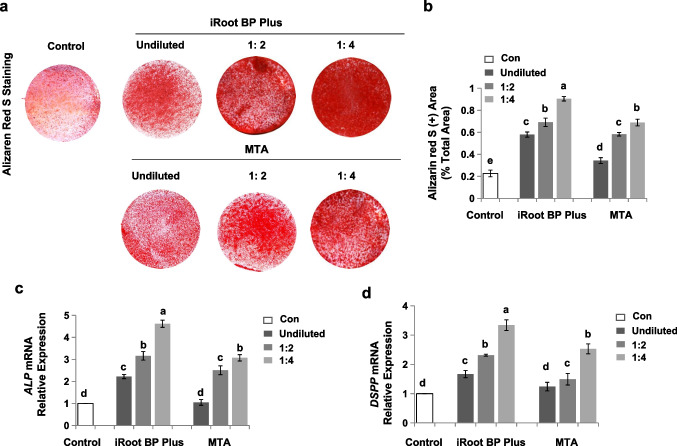
iRoot BP Plus enhanced the osteo-/odontogenic differentiation of SCAP. **(a and b)** iRoot BP Plus groups formed more noticeable mineral nodules than MTA and control groups in a concentration-dependent manner as assessed by Alizarin red S staining (*P* < 0.05). **(c and d)** iRoot BP Plus significantly elevated the expression of *ALP* and *DSPP* of SCAP at all doses, especially extracts at 1/4 dilution, relative to the MTA and control group (*P* < 0.05). Error bars: means ± SD

The odontogeneic potential of SCAP plays a critical role in pulp-dentine tissue regeneration and the continued development of the roots in immature permanent teeth [18]. Here, we used iRoot BP Plus, MTA, and control media to treat SCAP and then cultured cells under osteo-inductive conditions. As shown in Fig. 4a and b, iRoot BP Plus and MTA groups formed more noticeable mineral nodules than control group in a concentration-dependent manner as assessed by Alizarin red staining (Fig. 4a and b, *P* < 0.05). In addition, the formation of mineralized nodules was elevated in the iRoot BP Plus group relative to the MTA group (*P* < 0.05) (Fig. 4a and b). The result was also confirmed by the subsequent qRT-PCR assay. The *ALP* and *DSPP* mRNA level of SCAP were enhanced in the iRoot BP Plus group relative to the MTA and control group (Fig. 4c and d, *P* < 0.05). iRoot BP Plus significantly elevated the expression of *ALP* and *DSPP* of SCAP at all doses, especially extracts at 1/4 dilution (Fig. 4c and d, *P* < 0.05). These results demonstrated that iRoot BP Plus exerted great osteo-/odontogenic potential in a concentration-dependent manner.

### iRoot BP Plus promoted SCAP-based pulp-dentine complex regeneration in vivo

**Fig. 5:**
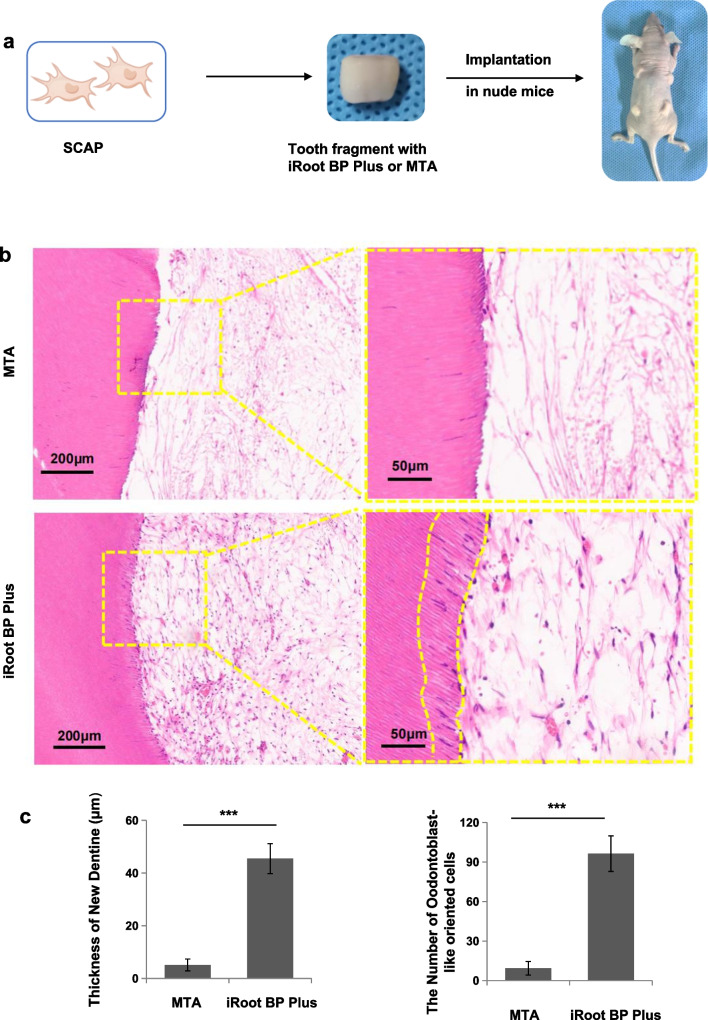
iRoot BP Plus promoted SCAP-based pulp-dentine complex regeneration in vivo. **(a)** The images showing the procedure of tooth slice with SCAP implanted subcutaneously on to the backs of immunodeficient mice. **(b)** HE staining showed that there was a new formed continuous dentine layer (dotted line) and lots of odontoblast-like oriented cells, bursting into dentinal tubules. The dental pulp fibroblasts were interwoven mesh in the root canal and lots of angiogenesis in the iRoot BP Plus group. **(c)** Quantiative analysis showed that the thickness of the new dentine and the number of odontoblast-like oriented cells in iRoot BP Plus group were significantly increased when compared with the MTA and control groups (*P* < 0.001). Error bars: means ± SD

To determine the in vivo capacity of inducing pulp-dentin complex of iRoot BP Plus as a coronal sealing material, we constructed a subcutaneous tooth slice implantation model. The procedure was shown as Fig. 5a. The tooth fragments were harvested after a 2-month implantation. H&E staining showed that a new continuous dentine layer was regenerated in the iRoot BP Plus group, in which considerably more odontoblast-like oriented cells were uniformly distributed over the pre-dentine border, thus forming odontoblast-like oriented cells in the dentinal tubules (Fig. 5b). However, an obvious pre-dentine and odontoblast layer was not observed in the MTA group (Fig. 5b). In addition, the dental pulp fibroblasts appeared as an interwoven mesh in the root canal in both the iRoot BP Plus and MTA groups, whereas the collagen fibrillar network was more compact and more obvious vascularization was found in the iRoot BP Plus group. (Fig. 5b). The thickness of the new dentine was significantly elevated in iRoot BP Plus group when compared with the MTA group, and the number of odontoblast-like oriented cells in iRoot BP Plus group was higher than MTA group (*P* < 0.001) (Fig. 5c and d). Taken together, these experimental evidences suggested that iRoot BP Plus promoted SCAP-based dentine-pulp complex regeneration in vivo.

## Discussion

REPs represent a new strategy for the treatment of immature teeth with necrotic pulp. Based on the triad of tissue engineering concept, REPs are aimed at inducing stem cells and growth factors based on blood flow from the periapical tissues into the chemomechanically debrided canal space of teeth for possible pulp tissue regeneration [18]. In REPs, a tight coronal seal can isolate the root canal from the external surface of the tooth and prevent reinfection, which is beneficial to pulp tissue regeneration [3]. Therefore, a good coronal seal is a crucial factor for achieving a favorable prognosis. An ideal coronal sealing material would adhere and adapt to the walls of the cavity, prevent the leakage of micro-organisms and their toxins into the cavity, and have dimensional stability and biocompatible properties [3]. SEM examinations represent a suitable method for assessing marginal adaptation because of their high magnification and fine resolution [20]; therefore, it was used to determine the marginal adaptation in the present study. Our studies found that the marginal adaptation of iRoot BP Plus was better than that of MTA, which may be related to the setting properties of MTA. According to the manufacturer’s instruction, MTA must be mixed with sterile water to reach a sandy consistency [21, 22]. In addition, the addition of bismuth oxide decreases the marginal adaptation ability of MTA by destroying its mechanical stability [23]. Compared with MTA, the finer particles and dimensional stability during setting of iRoot BP Plus may lead to a better sealing ability [24]. Thus, iRoot BP Plus had a better marginal adaptation ability than MTA. In addition, some studies reported that EDTA was able to interfere with the hydration reaction during MTA solidification, resulting in the decreased physical properties and poor biocompatibility of MTA [25–27]. The above findings indicated that, in REPs, 17% EDTA as the final rinsing might lead to the untight adhesion of MTA and dentin. And our study also found that there was an obvious void between MTA and dentin by SEM observation, but not between iRoot BP Plus and dentin, providing evidences for iRoot BP Plus as a promising coronal seal material in REPs.

We aimed to simulate the clinical circumstance of REPs in which the contact between SCAP and coronal sealing material occurred indirectly through blood clots [18]. The concentration of material components in contact with SCAP may be gradually decreased, because the soluble components are continuously diluted in physiological fluids during the coagulation reactions [18, 28]. Thus, conditioned mediums of the iRoot BP Plus and MTA at various concentrations were prepared in this study. SCAP possess self-renewal and multipotent differentiation properties [15]. Since SCAP enter the root canal with blood in REPs, SCAP have been considered to be the most critical cell source for pulp-dentine regeneration [17]. Therefore, excellent biocompatibility with SCAP is an essential prerequisite of successful coronal sealing material used in REPs. Cell proliferation is important characteristics for assessing the biocompatibility of dental materials, so Brdu labeling and MTT assays were performed to evaluate cell proliferation in this study. The proliferation rate of SCAP in iRoot BP Plus groups were not significantly different relative to that of the MTA group or control group on days 1, 3, 5, and 7. These results indicated that iRoot BP Plus is a safe coronal sealing material without cytotoxicity. By contrast, studies have reported that iRoot BP Plus slightly suppressed the viability of dental pulp cells relative to that of the MTA group [29], and Zhu et al. reported that the iRoot BP Plus promoted the viability of dental pulp stem cells (DPSCs) [12]. These inconsistencies may be related to the different cell lines, stimulation modes, and setting times applied for the test materials.

Based on cell homing theory, REPs achieve pulp tissue regeneration by modulating the migration and differentiation of endogenous stem cells residing around the root apex [18]. Endogenous progenitor cell migration is one of the prerequisites for pulp-dentine regeneration in REPs [30]. iRoot BP Plus has been shown to accelerate the cellular migration of DPSCs [12, 31]. In this study, we further found that iRoot BP Plus enhances the migration of SCAP in a concentration-dependent manner, which may affect the next phase of the interaction, which involves odontoblastic differentiation and mineralization, and determine the final curative effect of REPs. In addition, our results revealed that diluted extracts of iRoot BP Plus also promote SCAP migration compared with the MTA and control groups, which suggested that when the bioactive components of iRoot BP Plus infiltrate into the apical, they still have a strong ability to recruit SCAP into the root canal. However, limited information is available on the detailed mechanisms underlying iRoot BP Plus-mediated SCAP migration; thus, further investigation is required.

An ideal coronal sealing material should promote osteo/odontogenic differentiation of stem cells homing to the root canal, which resulted in the regeneration of pulp-dentin complex. As calcium silicate-based biomaterials, both iRoot BP Plus and MTA had stronger effects on promoting osteo/odontogenic differentiation through Ca and Si ion release [29, 32, 33]. Furthermore, the release of Si ions and the associated alkalinity inhibits osteoclastogenesis and bone resorption [34]. In the present study, both iRoot BP Plus and MTA had a superior promotion on the osteo/odontogenic differentiation of SCAP than the control group, and iRoot BP Plus had a significantly stronger capacity, which is consistent with previous studies [29, 32]. In addition, our results revealed that low-concentration extracts of iRoot BP Plus presented significantly higher osteo/odontogenic differentiation of SCAP compared with the other groups, which suggested that iRoot BP Plus may also promote the osteo/odontogenic differentiation of SCAP at the apex, thus inducing apical barrier formation and promoting root development. What’s more, in this study, we evaluated the in vivo osteo/odontogenesis of SCAP and confirmed that iRoot BP Plus had a good promotion on the formation of SCAP-based pulp-dentin complex in vivo. These data demonstrated that iRoot BP Plus presented a good ability to boost the osteo/odontogenic differentiation of SCAP.

Furthermore, calcium hydroxide as the main component of MTA and iRoot BP Plus, when it reacts with body fluids, will turn it into an alkaline medium [35]. High alkaline pH has been reported to alter the integrity of the cytoplasmic membrane and microbial activity [36]. According to the results of our experiment, the pH value of iRoot BP Plus and MTA did not significantly differ, which may suggest that iRoot BP Plus has similar antibacterial properties as MTA [37]. Moreover, iRoot BP Plus overcomes the drawbacks of MTA. Unlike MTA, iRoot BP Plus is ready-to-use, more clinically operational, and aluminum-free, and it apply tantalum and zirconium oxide instead of bismuth oxide as a radiopacifiers; therefore, harmful components are not released after curing and tooth discoloration does not occur [38]. Therefore, iRoot BP Plus is considered a promising alternative to MTA as a coronal sealing material in REPs.

As far as we know, the current reports of iRoot materials mainly focus on iRoot FM. Investigators demonstrated that iRoot FM showed a satisfactory antibacterial property without cytotoxicity to SCAP, so that can be used as intracanal medicaments for endodontic treatment [39]. Moreover, our previous study showed that iRoot FS improved the osteo/odontogenesis of dental stem cells and had a potential application as an apical sealing material in apexification [40]. However, the biological basis of iRoot BP Plus as a crown sealing material for REPs remains unclear, especially the effects of iRoot BP Plus on SCAP. In this study, we set the concentration gradient of material extract to simulate the clinical circumstance of REPs, and studied the effects of iRoot BP Plus on the entire cellular responses of SCAP. In addition, we further verified that iRoot BP Plus can promote pulp-dentine complex regeneration in vivo*.*

## Conclusion

iRoot BP Plus showed excellent sealing ability and promoted the migration and osteo/odontogenesis of SCAP without cytotoxicity. Furthermore, iRoot BP Plus promoted SCAP-based pulp-dentine complex regeneration in vivo. Thus, the results of the present study provided new experimental evidences for the clinical application of iRoot BP Plus as a promising coronal sealing material in REPs.

### Supplementary Information

Below is the link to the electronic supplementary material.

Supplementary file1 (PDF 272 KB)

Supplementary file2 (PDF 339 KB)
