# Supplementary material for: In vitro and in vivo evaluation of iRoot BP Plus as a coronal sealing material for regenerative endodontic procedures
Source: Clin Oral Investig. 2024 Jan 3;28(1):70. doi: 10.1007/s00784-023-05468-3 (PMC10764398; doi:10.1007/s00784-023-05468-3)
Supplement: Supplementary file 1 — Supplementary file1 (PDF 272 KB) [file 784_2023_5468_MOESM1_ESM.pdf]

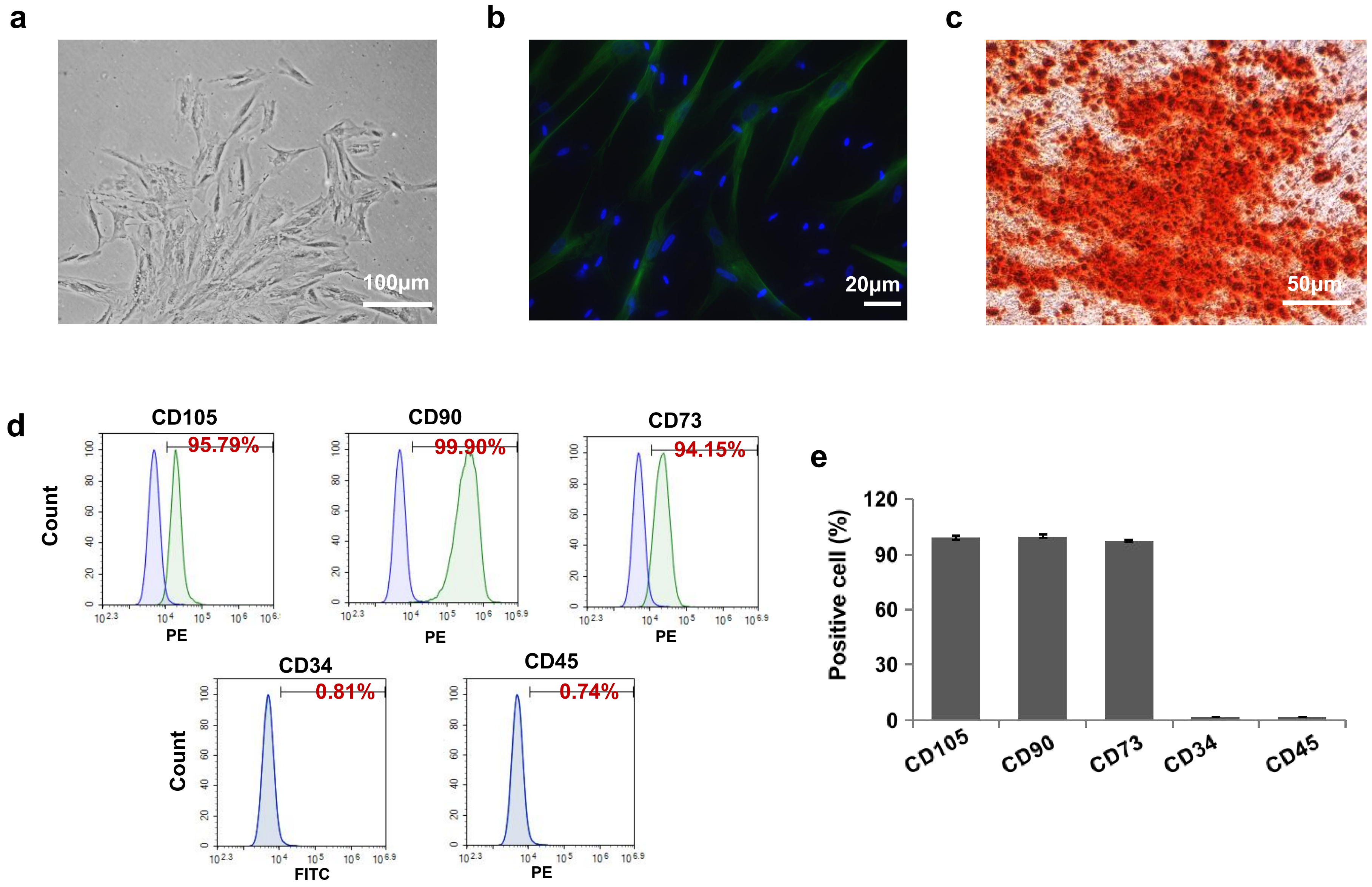

Supplementary Figure 1. Characterization of SCAP. (a) SCAP retained a spindle shape under the inverted phase contrast microscope. **(b)** SCAP differentiated into neuron-like cells, and expressed the neural cell marker βIII-tubulin. **(c)** Alizarin red S staining showed that SCAP formed mineralized nodes under osteogenic induction. **(d and e)** Flow cytometric analysis showed that SCAP expressed mesenchymal stem cell surface markers (CD105, CD90 and CD73), but failed to express hematopoietic stem cell markers (CD34, CD45).
