# Supplementary material for: In vitro and in vivo evaluation of iRoot BP Plus as a coronal sealing material for regenerative endodontic procedures
Source: Clin Oral Investig. 2024 Jan 3;28(1):70. doi: 10.1007/s00784-023-05468-3 (PMC10764398; doi:10.1007/s00784-023-05468-3)
Supplement: Supplementary file 2 — Supplementary file2 (PDF 339 KB) [file 784_2023_5468_MOESM2_ESM.pdf]

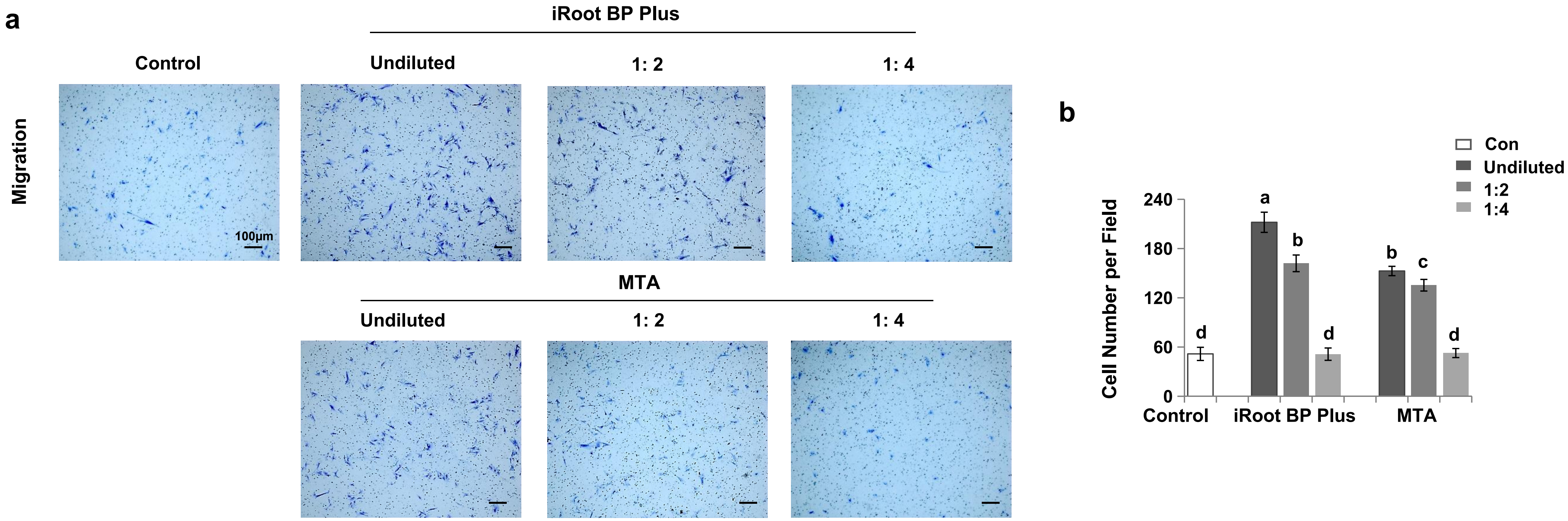

Supplementary Figure 2. iRoot BP Plus promoted the cell migration of SCAP. (a and b) Transwell cell migration assay showed that more migrated SCAP among the iRoot BP Plus treated SCAP than among the MTA treated SCAP at 24 hours in a concentration dependent manner ($P < 0.05$). Error bars: means ± SD.
